# Perceived Loneliness, Social Isolation, and Social Support Resources of Frail Older People Ageing in Place Alone in Italy

**DOI:** 10.3390/healthcare12090875

**Published:** 2024-04-23

**Authors:** Maria Gabriella Melchiorre, Marco Socci, Giovanni Lamura, Sabrina Quattrini

**Affiliations:** Centre for Socio-Economic Research on Ageing, IRCCS INRCA—National Institute of Health and Science on Ageing, Via Santa Margherita 5, 60124 Ancona, Italy; m.melchiorre@inrca.it (M.G.M.); g.lamura@inrca.it (G.L.); s.quattrini@inrca.it (S.Q.)

**Keywords:** frail older people, ageing in place, social support/resources, family, loneliness, mixed methods analysis, social isolation, well-being, Italy

## Abstract

This paper presents some findings from the IN-AGE (“Inclusive ageing in place”) study, which the authors carried out in 2019 in Italy. It explores the available social support resources for frail older people with functional limitations ageing in place alone, and possible links between their social isolation and perceived loneliness. The authors conducted qualitative/semi-structured interviews involving 120 participants aged 65 years and over, and used a mixed-methods analysis (quantitative/qualitative). The main results show the family as the main help resource for daily activities, but also for intimate confidences against social isolation, especially when said relatives live close. Family confidants are less present when seniors are supported by friends/neighbours or/and public services. Moreover, the family is valuable for decreasing loneliness, although not always. However, some older people feel particularly alone when they are supported by public services. Such a complex context draws attention on the need of support for frail seniors living alone and could provide insights for policymakers on adequate policies for preventing and managing loneliness and social isolation in later life. This is especially relevant when family (and other) resources are not available or scarce, also considering the opportunities offered by technology, which can help seniors remain socially connected to relatives, friends and their overall community.

## 1. Introduction

In Italy, as of 1 January 2023, older people over 65 years represented 24% of the total population, those over 80 represented almost 8% of the population and the demographic scenarios foresee a significant increase in the so-called “oldest old” demographic, i.e., over 80 and 90 years. Furthermore, 49.3% of seniors over 65 live alone [1]. Overall, 11.2% of older people aged 65+ have serious difficulties in performing at least one personal care activity, and 10.3% are not independent when bathing or showering. Other important difficulties for seniors with functional limitations regard domestic activities, with 30.3% having serious difficulties in carrying them out. This share increases up to 47% after the age of 75, especially concerning heavy domestic activities which require a certain physical autonomy, such as shopping. Also, a third of seniors with severe functional impairment live alone, reaching 42% among those aged over 75 [2]. The proportion of 65+ people in 2023 in Italy represents the highest value among European countries, with the European Union average being equal to 21.5% [3]. Moreover, in European countries in 2022, about 34% of seniors lived alone [4], and 52.2% of people aged 65 or over reported some disabilities [5].

This is to highlight that living alone in old age is associated with poor health status, and also to higher risk of mortality [6]. Living alone indeed impacts the frailty of seniors, which is associated with socio-demographic aspects (old age, low income), clinical conditions (e.g., multimorbidity), lifestyle (e.g., physical inactivity) [7,8], in addition to the absence of social support resources, i.e., social frailty [9]. Frailty itself can lead to disabilities, with possible admissions to hospital and care institutions [10]. It can reduce social interactions [11,12], with consequent social exclusion, low quality of life, economic deprivation [13], and possible both social isolation [14] and loneliness [15,16]. On the other hand, both social isolation and loneliness could increase the risk of becoming frail [17].

According to several previous authors [18,19], social isolation is an objective concept, that is, the occurrence of decreased social interaction and ties [20], and can be measured by a low number and frequency of contacts with one’s own social network, and a lack of social integration. Similarly, Sandu et al. [21] state that social isolation is measurable by the frequency of meeting with relatives, friends/neighbours and former work colleagues, particularly affecting older people. For other authors [13], social isolation also includes lacking relationships with family and friends/neighbours for intimate confidence (e.g., persons to whom to confide any concerns). It furthermore regards aspects such as their physical proximity, frequency, modality of contacts (face-to-face, by telephone) and reasons for confidence (to ask for practical/psychological support) about a need to be assessed. Menec et al. [22] indicate an additional distinction between the structure (e.g., number and frequency of contacts) and the function (social support) of the social networks. Fiori et al. [23] differentiated among structure, function and experienced quality of social relations and social networks, which are particularly crucial in old age, in this case with regard to seniors aged 70 to 103 years.

Loneliness conversely represents a more subjective condition relating to the perception of the scarce attention from, or lack of contacts with, other persons. Therefore, it is connected to feeling alone or neglected by family members and friends, who sometimes may seem little or not at all attentive to the intimate and social needs of their older cared for relatives/friends [20,24]. In other words, the older persons express a negative opinion with respect to the quality/quantity, or perceived value, of the emotional, practical and informational support provided by others. They could indeed record an absence of meaningful/satisfactory relations, also with regard to the loneliness thresholds, i.e., different overall “expectations” towards family members [13,25,26]. Thus, loneliness is the experience of an inconsistency between real and desired social relationships [20]. Furthermore, the authors of [27,28] specified that loneliness and social isolation are linked to poor health outcomes, in particular with the former representing a possible risk factor for depression, and the latter a potential risk factor for physical inactivity and also mortality. Other authors found a positive relation between ageing and loneliness, especially in very advanced age [29,30,31], since social contacts in later life are hampered, e.g., by the death of peers and functional/mobility limitations.

Ageing in place depends, however, on the available supports [32,33]. Care arrangements for older adults differ across Europe, with strong informal/family care and intergenerational co-residences, which are available mainly in southern European countries, and formal/institutional care services mainly in northern ones [34]. Moreover, in Northern and Western Europe, in recent years, the highest expenditures on long-term care (LTC) were registered, whereas southern (e.g., Italy) and central European countries had the lowest ones [35]. Also, in Europe, homecare is adopted by about 22.2% of older people aged over 65 years who live in the community and need LTC [35]. However, informal care amounts to 80% of the overall LTC provision in the “old Continent” [36]. With particular regard to Italy, which is a traditional familistic country, apart from 18.8% of seniors who declare not receiving adequate help in relation to their daily life needs [37], in 2019 especially family members provided help to seniors with personal care or household activities. Over 50% of them indeed receive support from relatives, 17% make use of paid staff and 6.4% receive help from other people (e.g., friends, neighbours, volunteers). Among older people who have serious difficulties in personal care, help from relatives (cohabiting or not) goes up to 84.4% [38]. Moreover, 73% of seniors living alone, and 93% of those living in another type of household (e.g., with cohabiting relatives), receive help from family members. Also, the former use both paid services (e.g., for housework) and a private/paid personal care assistant (PCA) more than the latter (respectively, 44% and 31% vs. 28.6% and 21.3%) [38].

In Italy, to live at home for as long as possible, at least with a PCA, vs. institutionalisation, is also the preferred housing solution of seniors, who show overall little intention to move to a nursing home (also in the light of its high cost), which is defined by most of them as a place of loneliness itself, or to live with children [16]. However, the decreasing overall family help (with children remaining the main care pillar), the high cost of PCAs and of other private home services (e.g., domestic home help) and insufficient public/professional home services (as for geographical coverage and intensity, i.e., few hours a week) all influence the effective and realistic possibility to “remain at home” in this country [36]. Also, even though friends and neighbours are sometimes an important source of help, in addition to volunteering associations, the former are often older persons themselves, and the latter represent a support that is not so widespread [36]. It is worthy to highlight that relatives, but also friends/neighbours, are important support for older people in expressing and reporting their necessities to physicians and other health professionals [13]. Such a context needs to be studied more in depth, for the complex implication impacting both social isolation and loneliness of frail older people living alone. These are crucial issues for researchers and policymakers, also following the current population’s rapid ageing.

In light of these considerations, this study proposes some findings from the “Inclusive Ageing in Place” (IN-AGE) research project [39], as the authors explained better in Section 2. They explored how frail older people ageing in place alone in Italy are supported in their daily life, and how they perceive loneliness and experience social isolation, by means of the following research questions: (1) What are the main family/social support resources for frail older people in carrying out activities of daily living? (2) What relationship do care networks have with social isolation and the loneliness of seniors living alone? (3) What relationship exists between the degree and type of social isolation experienced by older people and the level of loneliness they perceive? The authors suppose that ageing in place for as long as possible represents a key component of the quality of life, and a lack of support (whether material, e.g., for doing housework, preparing meals, providing transportation; or psychological, e.g., provision of emotional support and social relationships) represents an obstacle in this respect. Also, frailty impacts the social relationships/contacts of older people, which are important channels for seniors to communicate/confide their own both material and psychological needs. This situation could potentially in turn lead to social isolation and loneliness. The authors overall hypothesise that social support may thus buffer both isolation and loneliness. They also expect that older people, who are supported by relatives living close/in proximity, and have in-person relationships with family confidants, may feel less alone and less isolated. However, this could not happen, due to the crucial role, as also discussed in the literature, played by the expectations that older people have regarding social relationships. Thus, older people may be “objectively” isolated but do not perceive loneliness, and vice versa [13].

The results of this analysis could be useful for preventing and managing the conditions of limited autonomy of the ageing population. This is in consideration of the high risk of insufficient available assistance, especially for frail older people living alone, for whom family networks could however represent the main antidote in order to feel supported, not isolated and not alone. These findings could thus provide insights for policymakers on dedicated care strategies for supporting frail seniors, and preventing, or at least managing as best as possible, their loneliness and social isolation.

## 2. Materials and Methods

### 2.1. Study Design and Participants

The authors carried out the IN-AGE survey in three Italian regions (Lombardy, North; Marche, Centre; and Calabria, South) in May–December 2019, and involved 120 older people. They recruited 40 participants in both one medium-sized urban site (24 units) and one rural/inner area (16 units) in each region. They also selected the most vulnerable urban districts, such as degraded and poorly served peripheral areas [40], and defined the inner area as a zone with increasing ageing process, growing depopulation and few health–social services, especially for older people [41].

The authors used a purposive (not probabilistic) sampling, wherein participants had characteristics allowing for an adequate exploration of the study topics [42]. The inclusion criteria were the following: men and women aged 65 years and over; living alone at home without cohabiting family members, or with a PCA (living in or on hourly basis); intermediate mobility between limited/reduced within the home, and outside the home with help; absence of cognitive impairment (in order to be able to answer questions independently); and absence of help by close family members (living in the same urban block/rural building) [39].

The local sections of Auser (voluntary association for active ageing, i.e., one of the main Italian association involving older people and providing help to seniors), professionals/operators of municipal/public home care/social services (SAD in Italian, i.e., Servizio di Assistenza Domiciliare) and other local/voluntary associations (Anteas, i.e., National Association All Ages Active for Solidarity; Caritas, i.e., pastoral body of the Italian Bishops’ Conference for the promotion of charity) supported the recruitment of participants. The territorial contacts preliminary verified the eligibility of the latter, based on the inclusion criteria fixed for the study (initial screening/pre-interview). In particular, they verified the cognitive status and autonomy (as intermediate mobility) of seniors, based on their own information and assessment, with further confirmation by relatives and interviewers. These operators then provided seniors with a detailed information letter on the aim and procedure of the survey, in order to collect their possible willingness to participate as preliminary adhesion. The operators then communicated the names and contact details of the potential respondents (address and telephone numbers) to the research teams. In this regard, they also obtained a first verbal consent from seniors to be interviewed, and to pass on their personal details to researchers. Overall, 88 (42%) out of 208 involved seniors declined, due to occurred illness or other reasons, e.g., second thoughts about interview concerning personal issues, despite a first positive adhesion.

### 2.2. Instruments, Data Collection, and Ethical Issues

The authors administered semi-structured interviews containing 55 questions, of which 21 were close/quantitative and regarded socio-demographic characteristics, physical/functional limitations in daily living activities, type of mobility (inside/outside the home) and main types of available social support resources/care arrangements (family, friends, neighbours, services). Moreover, 34 open-ended/qualitative questions explored more in depth the following dimensions: overall family context/support (e.g., composition of the living family/parental network and their housing proximity); characteristics of supports (e.g., frequency/intensity of help); loneliness (e.g., feeling alone/neglected/isolated from others) and social isolation (e.g., typology/frequency of contacts with family/friends/others).

Overall, the authors addressed the topics with questions adapted from previous studies [43,44], and with some inputs from items contained in other research instruments, all contributing to set up a preliminary conceptual framework. This allowed in turn to build the topic guide also by means of theoretical-based definitions, which already emerged from the literature. In particular, they only assessed the level of physical/functional limitations by using standardised tools. Conversely, they addressed social support (apart from an initial general close question on main four types), social isolations and perceived loneliness, with reworked/ad hoc open questions (also following the qualitative orientation of the study). Functional limitations included thus 12 Basic and Instrumental Activities of Daily Living (ADLs-IADLs) [45], however integrated with two mobility limitations (going up/down the stairs and bending to pick up an object), in addition to sensory limitations in hearing and seeing [46,47]. In particular, ADLs assess very basic activities necessary for survival, and which allow a person to take care of themself, whereas IADLs are more complex ones, necessary for independent living. ADLs are the following: getting into/out of bed, sitting/rising from a chair, dressing/undressing, washing hands and face, bathing or showering and eating/cutting food. IADLs include the following: preparing food, shopping, cleaning the house, washing the laundry, taking medication in the right doses and at the right times and managing finances. The authors addressed social support by asking some information about the persons who help the seniors the most (e.g.,: “Please, tell us about the person—relative, friend, neighbour, service operator—who helps you the most”; “Approximately, how often does this person help you in a week?”; “How far away does he/she live?”). They also explored social isolation by asking interviewees to talk about the persons (within their family, friendship and neighbourhood networks) to whom they confide any intimate concerns, and ask for practical/psychological support, or just for talking. Also, they asked the methods (e.g., face-to-face or by telephone) and frequency (e.g., daily/weekly/monthly/annual) of the meetings and contacts [13]. Moreover, the authors explored perceived loneliness by means of some general open questions, e.g., “Do you feel alone/abandoned?”; “How much does it seem that others are attentive to what happens to you?”. This paper focused on the quantitative aspects cited above, and the answers to some open questions regarding social support resources, social isolation and loneliness. Three psychologists and three sociologists (two for each region, on the whole five females and one male) with deep expertise in quantitative/qualitative data collection, conducted face-to-face interviews (lasting about 60–90 min) at the seniors’ homes. They also audio-recorded and transcribed in full/verbatim (from audio to electronic text format) the narratives, following a scheme/protocol that essentially re-proposed the outline of the interview. Before conducting the interviews, the interviewers participated in a methodological training seminar, regarding aim and protocol of the study. They also realised a pilot test by conducting three test interviews (one in each region) that made it possible to refine and improve the preliminary thematic framework. On the whole, the interviewers produced 280 pages of transcriptions for the analysis.

Before starting the data collection, the Ethics Committee of the Polytechnic of Milan approved the study (POLIMI, Research Service, Educational Innovation Support Services Area, authorization No. 5/2019, 14 March 2019). Moreover, all participants signed a written informed consent form, and were reassured on the anonymity, privacy and confidentiality of their personal information provided, according to ethical issues indicated by the European General Data Protection Regulation (GDPR) No. 679, of 27 April 2016 [48]. In particular, the authors used codes to replace the respondents’ sensitive information (i.e., name, address and telephone number).

### 2.3. Data Analysis

The authors performed an overall mixed-methods analysis (mainly descriptive) of the 120 interviews, without distinction for regions. They summarised the quantitative data, which were collected by means of close questions (e.g., socio-demographic), by using Microsoft Excel 2022 (Microsoft Corporation, Washington, DC, USA) and by calculating the related percentages (univariate and bivariate analyses). They followed five standard phases of the framework analysis technique [49] to provide the qualitative analysis of open answers, as follows: in-depth reading of transcribed interviews, identification of macro/sub categories, identification of codes, construction of the thematic charts (two-way matrices, with rows corresponding to cases/interviewees and columns to categories), where to break down the narratives and interpretation of results [50].

After reading the transcriptions, and as preparatory step for the qualitative analysis, the authors also constructed an overall list of main emerged categories and possible labels, also confirming and integrating the initial conceptual framework. All the research units then collectively discussed this “tree” and used it to build the thematic charts (one for each category and possible sub-categories, further split between urban and rural sites) by means of Microsoft Excel sheets, as a useful template for reducing and ordering the statements emerging from the interviews [42]. In particular, six researchers (two for each region, M.G.M and S.Q. for the Marche region) broke down 20 transcriptions each in the charts. Then, for this paper, M.G.M. and M.S. analysed all the charts transversally, i.e., with regard to some aspects/themes closest to their own expertise (e.g., respectively, characteristics of supports for the former, and social isolation and loneliness for the latter). They conducted the overall qualitative analysis with a step-by-step procedure, that is, firstly by processing the results regarding single sites (three urban cities and three rural/inner areas) and then by capturing emerging similar/different patterns as a whole. Finally, the authors discussed all together the appropriateness of both reading and labelling the contents. In this respect, they ensured a close dialogue, between the researchers and interviewers, by means of an iterative process.

The authors moreover carried out a thematic content analysis to identify the relationships and links among the themes which emerged [51]. They performed this manually, without using a dedicated software, as made possible also by the literature [52,53]. They preferred a manual management of the findings since qualitative analysis represents a critical and repeated thinking process, requiring a deep/line-by-line reading of the interviews. This approach allows researchers to become progressively much familiar with the narratives, by exploring the charts several times, with a continuous navigation among categories and labels, thus addressing possible new sub-categories and further relationships between them [42]. In other words, a manual analysis makes it possible to make connections more in depth than those provided by a specific software, which can however be a useful tool for a first storage/archiving and organisation of collected data. Moreover, the preliminary conceptual framework put in the topic guide, as well the step-by-step procedure of the analysis (which are both mentioned above), facilitated this manual assessment.

The authors moreover quantified the qualitative responses in order to provide a more precise picture of the seniors in terms of frequency counts of the statements. In particular, the identifications of qualitative accounts put in evidence the main categories to be then quantitatively analysed and presented in tables, as findings summarising and guiding the interpretation of recurring patterns of meaning [39]. Moreover, the authors elaborated some more articulated quantitative classifications in order to better present the counts of statements mentioned above, with regard to functional limitations, social support, social isolation and loneliness, as described below.

Regarding the level of physical limitations, the authors considered three degrees of difficulty, i.e., activity performed in autonomy, with help or not performed (i.e., the senior is “not able”). Then, they provided four grades of functional frailty: “mild frailty” when the older person is able to carry out all ADL-IADLs (no activity labelled “not able” is referred); “moderate frailty” when the older person is unable to perform one to two ADL-IADLs; “high frailty” when the older person is unable to perform three to four ADL-IADLs; “very high frailty” when the older person is unable to perform five or more ADL-IADLs [54].

Regarding social support, the authors computed only the share of family support, as a proportion of the total available help (from family, private services, public services, PCAs, friends/neighbours). They identified a minority of family support (number of relatives who help) when it was less than 50% of the total, and a majority when it was greater. Moreover, they assessed the number of family members living geographically close to seniors and who help them (physical proximity: who live in the same urban city/rural municipality of respondents). Conversely, they fixed (as already stated above) the presence of help by close family members living in the same urban block/rural building as an exclusion criterion.

The authors also analysed the objective dimension of social isolation only as a share of the family members within one’s overall confidential network of seniors (including friends and neighbours). They identified a minority of family confidants (number of relatives for confidences) when it was less than 50% of the total, and a majority when it was greater. Moreover, they considered only face-to-face contacts (with confidants in general and not just with regard to family members) because everyone reported contact by telephone for confidences.

The authors analysed the subjective perceived loneliness as overall presence–absence of this feeling, and then classified it into four levels. Absent/mild: if the person does not feel alone or rarely perceives a sense of loneliness. Moderate: if the person feels sometimes lonely, but this feeling is linked to contingent events, certain times of the day/week and certain times of the year (e.g., respectively: a rainy day, the night or the weekend, major holiday periods, i.e., Christmas and Easter), and it is almost never described as an intense sensation. High: if the person often feels alone and describes this feeling as intense. Very high: if the person often feels alone and this feeling is very intense and also results in psycho-physical effects (e.g., insomnia, depression and suicidal thoughts) [13,55].

The overall quantitative analysis presented in the tables shows both absolute and percentage values (n and %). Sometimes, both do not correspond to the respective totals (of participants and 100%), i.e., when more responses/statements of a single case were possible, or in the cases of missing/no statements. Also, sometimes the sums of the percentages did not correspond to 100% following the rounding of individual figures. Moreover, the authors quantified qualitative findings with a “qual to quant” approach, thus maintaining a qualitative dominant, with quantitative data not representing primary results with statistical appraisal [39]. Thus, they did not present *p* and SD values (respectively, significance level and standard deviation) in the tables.

The authors have integrated/supported the analysis of findings regarding social support resources, perceived loneliness and social isolation, by adding relevant quotations/verbatim statements which emerged in the transcription of the interviews [56]. They classified/coded each quotation with IT (for Italy) and progressive interview number (1–120). More information (regarding setting, sampling, measures and data analysis) is available in a previous publication [39], from which the authors partly drawn/adapted the present section Materials and Methods.

## 3. Results

### 3.1. Sample Characteristics

Table 1 shows that the respondents were mainly older people over 80 years, women, with low/medium education, widowed and living alone without a PCA. Above all, mild/moderate functional limitations, as well as mobility also outside the home, although with help, emerged. More information on the sample is available in the work by Melchiorre et al. [39].

### 3.2. Types of Social Support-Resources

The family (especially children) represents the greater support for older people with functional limitations to perform daily tasks (78%), followed by private services (e.g., domestic home help), friends/neighbours, public services (e.g., SAD) and PCAs (Table 2).

### 3.3. Social Isolation and Social Support Resources

Overall, the respondents reported having family confidants (66%). Moreover, the possibility—in case of need—of confiding in one’s own relative is greater when the share of family help on the total available support is greater (65%). Conversely, 73% report no confidant (at all, or familiar) and no family support (Table 3).

As revealed by narratives, the possibility to have family support also implies the opportunity to have confidants among relatives.

*I confide in my children, especially girls, who also help me in daily activities*.

(IT_64)

*I see my niece every day, every morning she comes. She helps me, I talk with her, she comes every day. If I need she never leaves me (…). She never neglects me. I am not isolated, never! I do not suffer.*

(IT_87)

*All my relatives come to visit me very often, cousins, grandchildren! They also help me.*

(IT_114)

*When I have some worries I talk to my brother, if I need something he is available.*

(IT_68)

Thus, some seniors who report having no family help, also report having no confidant.

*My brother and I talk very rarely. Some years ago we were more close, we helped each other. Currently he does not want to listen complaints. I cannot stand this indifference from him anymore*.

(IT_79)

*I cannot call my son since his wife does not want I do it. At least once she called me. Now nothing. I have no relationship with my son*.

(IT_88)

However, some respondents do not confide in their family members because they fear not being understood or making them worry and angry, or because they do not have a good relationship with them.

*I cannot tell too much to my daughters, since they do not always understand me*.

(IT_47)

*I cannot tell my son anything because he gets angry afterwards. He gets angry when I am sick*.

(IT_43)

*My sister has her family. We do not talk anymore because we clashed in a very violent way*.

(IT_95)

In some cases, the seniors prefer to not confide in anyone at all.

*I do not tell my intimate things to anyone, I keep them to myself. I keep my worries. I have never told anything to anyone*.

(IT_11)

When seniors are supported by at least one family member (94 units), these family members mainly also live close, that is, in the same urban city/rural municipality wherein the older person lives (60 units). With regard to possible links between the network of family confidants and the number of family members who help and are close, confidences are missing, or at most exist with a minority of family members, when no relative who helps, or at most only one, lives close. Otherwise, the possibility of confiding mostly/only in the family is greater when older people can trust the support of two/more close family members (38% and 32%, respectively) (Table 4).

From the interviews, the following verbatim sentences indeed emerged:

*I see my daughters very often, they help me, I can confide with them. They live close to me*.

(IT_87)

*I trust only on my nephew. I tell him everything. When my mobile phone freezes, I call him, if I have a financial problem, he makes me a bank transfer sometimes, but he lives in Spain. He calls me every day, but he lives far away*.

(IT_52)

With regard to the relation between seniors’ face-to-face contacts with their confidants in general (not just family members, overall two and more in 71% of cases), and family members who help and are close to them, the impact of geographical proximity is further confirmed. If the family members who help do not live close, there are one to two face-to-face contacts in 64% of cases, and even no contact for 12%, whereas almost 80% of older people report two and more contacts when two and more family helpers are living close (Table 5).

A greater presence of close family members who help seems thus to also imply a greater presence of face-to-face contacts.

*I have worries, many times, but I talk about them with my grandchildren, or with friends, who come to visit and help me. They live nearby*.

(IT_80)

When relatives who help do not live close, face-to-face contacts are scarce, or even lacking, and such a context can become very hard to manage.

*I am always at home, seeing these four walls, this makes me feel enormously melancholic. It’s like a prison*.

(IT_49)

This is, however, to highlight that, for all respondents, phone calls are overall reported as also a daily occurrence and thus very important to feel less isolated, especially when there are few opportunities for face-to-face contacts.

*I and my children talk every day by telephone. This is a beautiful routine for me*!

(IT_50)

*When I want to talk with someone, I have two friends who are available to listening to me on the phone. We talk every day*.

(IT_54)

With regard to other types of support (also combined), the presence of family confidants is more evident when help comes from a PCA (82%) and private/domestic services (66%). Otherwise, the absence of family confidants emerges more when seniors are supported by friends/neighbours or/and public services (40% in both circumstances). Also noteworthy is the total absence of confidants in 19% and 14% of cases of seniors receiving support from public and private services, respectively (Table 6).

Respondents highlight in particular how neighbours and friends are very important to feel less isolated, especially when family confidants are lacking or few.

*I have a neighbour who is also a friend, she helps me, we talk and confide together, we see each other every day from the balcony and on the stairs, she has the phone number of my children in case of emergencies*.

(IT_64)

*I have a very dear friend who helps me. We have known each other for 14 years. Our relationship is very close, we know our needs, we understand each other*.

(IT_44)

However, in later life, it is not frequent to still have friends who are alive.

*I have lost many old friends. Friends of my age are lost because they die and are no longer there*.

(IT_79)

Family confidants (but also overall confidants) are especially lacking when the support comes from public services.

*I have the support of a home care operator, who cleans my home. There is nobody else in my life, only God*.

(IT_91)

*I have the help of a social worker, and of a home care operator, who meet my needs for over 80%. It is so much. I have no other support or contact for confidences*.

(IT_50)

*I am fine with the support from public home services, especially when I am sick. I have to thank the public assistance so much, since I have no other person who can help*.

(IT_56)

Family confidants are conversely more present when a PCA (and private/domestic helpers) supports the seniors in daily activities.

*My daughters are very present, they help me for everything, even in managing the PCA. I tell them everything*.

(IT_64)

### 3.4. Perceived Loneliness and Social Support-Resources

Overall, the respondents mainly reported a mild/moderate level of loneliness (56%) vs high/very high levels (45%). The extent of support from loved ones seems to have its weight since seniors with overall family help, both minor and major, report an absent/mild sense of loneliness (respectively, 31% and 40%). Those who do not have help from their family report above all a moderate/high perception of this feeling (54%). However, 42% report serious levels of loneliness despite a greater family support (Table 7).

The presence and support from the family is thus very important to mitigate the perceived loneliness, and vice versa.

*I am fine, I do not feel alone, because my children call me and come all the time, they support me very much*.

(IT_64)

*My family feels my needs, even if I do not ask for anything*.

(IT_53)

*My son is always out doing his own things. My daughter is much more superficial, since her father died, she has her own life, I hear from her rarely. I feel alone*.

(IT_61)

*I am widow, I have never asked my children for anything, not even when my husband died. They also neglected me, they did not ask me if I had some need. I would have been happy if both children had said to me “do you need something mom”? It didn’t happen. I carry on everything with my own strength and I am very proud of this*.

(IT_45)

However, in some cases, the presence of a supportive family does not avoid feeling a high level of loneliness.

*I have relatives and friends who support me, but when it is evening I am alone. There is no evening without crying (…). There is loneliness even if you feel protected by people, by the attention of your children. I feel a lot the intimate solitude, which I do not show to anyone*.

(IT_44)

*I spend every Sunday with my daughters, but despite this I feel alone*.

(IT_55)

*I have the family but I miss a person I can trust on, to finally be able to say: ‘oh, I am relaxing, I have a “shoulder”, a trusted person I can turn to in case of emergency*.

(IT_54)

Also, with regard to loneliness, for all respondents, daily phone calls are fundamental to feel less alone. Some seniors would even like a dedicated telephone line for older people feeling alone.

*I do not feel loneliness, probably because I have two friends with whom I chat a lot by telephone. We are really chatty*!

(IT_42)

*Telephone contacts are very important for me. I spend a lot of time on the phone, the phone has always helped me a lot to feel less alone*!

(IT_54)

*Older people should have a telephone line ad hoc, especially when they would like to talk and nobody is available in person*!

(IT_52)

When the levels of loneliness and the number of family members who help and are living close are put in relation, 50% of seniors report a high/very high sense of loneliness despite being able to count on more than two close family members for support. Furthermore, a better context (no/mild loneliness) is reported equally (38% both) by those who have more than two close family members who give support and by those who have no close family support. However, in some cases, the total absence of close family support, or the presence of only one close relative who helps, is linked to severe loneliness (respectively, 41% and 38%) (Table 8).

Loneliness is thus really a personal perception, and the geographical proximity of supportive relatives for some respondents is important/fundamental, but not for all.

*My daughter lives in another region, very far away, this is heavy for me*!

(IT_47)

*My son now lives and works in the Philippines, one brother is deceased, another brother lives in another city. I have no relationship with him, since we see only at Easter and Christmas, twice a year. I have affections kept alive “at a distance”, almost virtually. The family members are all far away, they love me from afar, basically*.

(IT_52)

*My family does not live close to me but this is not crucial, my children are very careful. If we cannot see each other, we call by phone*.

(IT_64)

In other situations, however, the presence of supportive relatives living in proximity does not mitigate the loneliness.

*I feel alone even when I am with my family who supports me and lives close to me. I am widow and I feel the absence of my husband a lot*.

(IT_58)

Concerning other types of help (also combined), over half of the interviewees who are supported by a PCA (63%) do not suffer much from loneliness (absent/mild and moderate), as well as 58% and 56% of those who have support, respectively, from friends/neighbours and from private services (e.g., housekeepers). However, 53% of seniors suffer greatly from this condition when help comes from public services (Table 9).

The narratives depict all the different/opposed situations mentioned above well.

*I am never alone. There is always the PCA. I feel calm and safe*.

(IT_94)

*No, I do not feel alone. My neighbours and friends keep my company. We help and respect each other*.

(IT_105)

*I do not feel loneliness, since the domestic helper is with me great part of the day*.

(IT_67)

*I have support from public services but the quality of social relationships with the social workers is a problem, I have no social interactions with them*.

(IT_83)

*It is a bit of company that is missing (...). Loneliness is hard, especially on Saturday and Sunday, when public services operators are missing. It is painful to eat alone*!

(IT_50)

### 3.5. Social Isolation and Perceived Loneliness

From the analysis of the relation between social isolation and loneliness, a discontinuous trend emerged, with similarities between mild/high and moderate/very high levels of the latter (Table 10).

If the level of perceived loneliness is mild, there are many confidant family members (74%). At a moderate level, confidant family members are present but to a lesser extent (55%), and with a notable number of cases with no confidant (45%). If the level is high, the number of confidant family members increases again (71%). If a very high level is reported, again a notable number of cases report no confidant (48%), and the amount of family confidants decreases (52%)

In this respect, the overall narratives provide a mixed picture, with greater presence of family confidants both when light and serious loneliness is perceived.

*Mu children are careful, listen to me, help me, but everyone has to lead their own life. They have their life, I have mine. We spend time together especially at Christmas and Easter. I do not feel too much alone on the whole*.

(IT_2)

*I have my children to talk and for my needs but this is not enough. They have their own world, their own things. I do not feel supported, I miss my children! I feel really alone*!

(IT_93)

## 4. Discussion

With this study, the authors aimed to explore the social support resources of frail older people ageing in place alone in Italy, and possible links with their social isolation and perception of loneliness. The family emerged as the greater help also for intimate confidences, with face-to-face contacts, especially when they live close to the senior cared for. Family confidants are, however, more absent when seniors are supported by friends/neighbours and/or public services. The presence of the family, in particular when geographical proximity is possible, is also beneficial for mitigating loneliness, although not always. Moreover, some seniors feel alone when help comes from public services. Such a context highlights the need of supporting frail seniors living alone, in order to prevent and manage their loneliness and social isolation, especially when family resources are scarce, as explained in more detail below.

### 4.1. Networks of Confidants and Supports

The majority of older respondents reported activities of daily living they were unable to carry out, and the family represented their primary source of help, in addition to complementary supports such as services, friends/neighbours and PCAs, as confirmed by the literature [57]. The share of family help that is available for carrying out daily activities seems to also affect the possibility of having family confidants, i.e., a greater “material” help from family members implies in some way a greater “emotional/psychological” support, and perhaps also a lesser sense of isolation. According to ISTAT [58], the perception of the social support network (defined as physical and psychological support) registers a weak level of 18% and a strong level of 26% among the seniors over 65 years of age, while the respective values among those aged 15–34 are 13% and 33%. More recent data [59] highlight that, in 2022 in Italy, satisfaction with family and friend relationships was highest among young people, whereas it declined slightly in subsequent ages, reaching the minimum level among single men aged 55–74 years. The possibility of having an expanded support network shows a similar decreasing trend, reaching the lowest value among people aged 75 and over. Again, according to ISTAT [60], the time spent with other people also decreases in old age, with a clear variation between the two extremes represented by children aged 6–13 years (approximately 12 h a day) and seniors aged over 75 (seven hours). Moreover, older people who live alone, without co-resident family members, have contact with other people for only (about) four hours a day. Overall, several previous authors indicate that less socially isolated seniors are those with greater family assistance, in particular children who support them for daily needs and emergencies, thus having a fundamental role in alleviating isolation for older people, especially if they live alone [31,61,62]. Some authors found, however, that the availability of a dense/mixed network of social relationships (not only relatives) for frail seniors living alone, to interact and confide in each other, is fundamental to allow them to continue living in their own home, thus limiting their social isolation [13].

Based on our findings, a greater presence of close family members who help also seems to imply the possibility of confiding mostly/only in the family, with a greater presence of overall face-to-face contacts. The geographical/physical proximity of family members who give support (who live in the same urban city/rural municipality of the cared for senior) is thus of considerable importance for the actual assistance provided in everyday life, as both material and psychological support. Proximity seems to affect the possibility of relating in person with the family, as well as the possibility of having greater support. The literature also highlights how the geographical proximity of family members is overall crucial, so that in some cases care solutions with temporary/seasonal/emergency closeness between older cared for seniors and family caregivers are arranged [39]. Additional authors stress how conversely the physical distance, in particular between parents and children, negatively impacts the frequency of face-to-face contacts and the overall help received [63]. However, results from other studies [64] also suggest that the existing emotional support from relatives who are living in proximity could not be enough to mitigate the social isolation of seniors living alone. This happens since closeness itself could also lead to excessive and distressing contacts, even though generally physical distance hampers keeping regular and relevant interactions.

With regard to other types of support, our interviewed reported a greater presence of family confidants when they had the support from a PCA and private services, e.g., domestic home helper. When seniors are supported by friends/neighbours or/and public services, e.g., SAD, a more evident absence of family confidants emerged. It is likely that the family is overall more present when there is the necessity to check and manage an external/paid help, also with regard to contracts and financial aspects. Conversely, the family is more absent when the support is delegated to neighbours/friends and public services, these representing important references points for seniors, especially when the family is living too far/in another city or region and/or cannot help. The literature supports the considerations mentioned above and highlights how a division of care tasks is implemented between the family and PCA/domestic home helper, with the more physical and onerous “hard” care tasks “outsourced” by the families (e.g., personal care/hygiene, house cleaning). The latter remain responsible for “softer” but crucial functions concerning supervision, handling of administrative/bureaucratic/financial practices, relationship with doctors and shopping [65]. This context is also defined by the literature as the “crowding-in effect” [66]. Differently, friends/neighbours and public services seem to intervene more when family help is lower, or even absent. It is likely that, in these situations, the family, when it exists, is perhaps made up of a few members with poor care capacity/availability, and thus the relatives rely on the support of services, e.g., SAD, friends and neighbours. Moreover, according to the eligibility rules for accessing public services, usually the users are older people with a very weak overall care network, with both severe physical limitations and social fragility [67]. In this case, however, the so-called “crowding-out effect”, in particular between family and public care services, is not realised, since it occurs when more supportive LTC systems (e.g., in northern European countries) really relieve the family networks [66].

### 4.2. Perceived Loneliness and Support Networks

For our respondents, the support from relatives seems to mitigate the perceived loneliness, and vice versa. However, sometimes the presence of a supportive family does not impede one from feeling extremely alone. All this proves the very subjective perception of this feeling, which can make the seniors feel alone, even when immersed in a dense family support network [26,68]. This also takes into consideration the concept of loneliness thresholds, i.e., different subjective “expectations” with regard to social support by family members [13,25,26]. In this respect, some of the literature [21,69] specifies that social/cultural rules/values concerning family obligations impact individual expectations, with the latter being higher when family ties are stronger, e.g., in southern Europe (such as Italy), where relatives mainly provide social support networks for seniors. Thus, they may “have stronger feelings of loneliness when their relationship with family members deteriorates” [21] (p. 6). It should also be considered that not having/losing a spouse/partner, which is a frequent event in later life, significantly and negatively impacts loneliness [70,71]. Moreover, Yazawa et al. [72] found a positive association between being lonely and risk for hypertension, despite high levels of social support.

The fact that loneliness is a personal perception is also supported by our finding regarding the geographical proximity of supportive relatives, since this circumstance does not always seem to alleviate the sense of loneliness. One can indeed feel alone even with several family members who help and are close, if the multitude and closeness do not correspond to an effective quality/affection of care. Several authors found that family (especially children) proximity and frequent in-person contacts can reduce the loneliness of the older person cared for [73,74]. Other authors, however underscore that it is extremely difficult to assess and understand loneliness, since it can occur even in the presence of close/nearby contacts, if these do not represent ”meaningful” social relationships. Thus, even though lacking/scarce social close contacts can lead to loneliness, it is also possible to live alone and not feel loneliness [75]. Further study findings indicate that emotional loneliness (feeling of loneliness) may be more sensitive to changes in the close social network, whereas social loneliness (due to few social interactions) is less sensitive to external interactions with others [76,77]. Moreover, the “fluctuating nature” of loneliness should be considered as a temporary feeling that can even occur in some daily circumstances, e.g., in the evening/night and during the weekend, when people suffer most from being alone [76,78].

Concerning other types of support, loneliness is absent/mild and moderate mainly when help comes from PCAs, friends/neighbours and private services (e.g., housekeepers), whereas a greater loneliness seems to emerge when public services support seniors in daily activities. Regarding PCAs, private domestic home helpers and public professional home care, the same mechanism that the authors highlighted above for social isolation could work. Loneliness could indeed be attenuated by a “collaboration” between family and private external help, this implying a greater presence of the former [65,66]. In case of support from public services, the fact that eligible users are older people with an overall poor care network could play a role in fostering a sense of loneliness [67]. Concerning friends/neighbours, on the one hand when they help seniors the family confidants are less present, and on the other hand the presence of the former could contribute to alleviate the perceived loneliness, thus confirming their great importance for older people. Several studies indicate that friendship/neighbourhood networks are fundamental since they are often based on relationships which are built over time. The seniors turn to friends and neighbours so as not to disturb their family with too many requests in case of need. In this regard, the proximity of the respective homes, especially with the neighbours, facilitate such important contacts daily [60,79]. When people get older, the “extended” and “variegated” social network (relatives, friends and neighbours) decreases, while “exclusive” networks expand, e.g., those made up only of relatives or only neighbours. Neighbourhood relationships in particular intensify, since they can meet the support needed in everyday life and also alleviate loneliness [60].

It should moreover be underlined that help from public services is perhaps more impersonal/professional and aseptic than other types, although not always. Even the usual methods of providing SAD in Italy seem to have an impact on loneliness. A few and insufficient hours of assistance (national average of 8.2 weekly hours for the user) [80], in addition to the frequent rotation of operators, certainly do not facilitate the creation of friendly relationships with the older user. Also, from a previous study on the experiences of social and health professionals, in dealing with situations of loneliness among seniors [81], it emerged that sometimes the latter, when experiencing such a feeling, also tend to refuse the support of the operators, as if it was not enough. This seems to be attributable to the suffered and not replaceable absence of the family.

### 4.3. Social Isolation and Loneliness

The relationship between social isolation and loneliness emerging from our results do not indicate a clear increasing or decreasing trend, or a linear link, i.e., higher loneliness and lower number of supportive family confidants or vice versa. Thus, loneliness may or may not be associated to a lack of confidants. However, the literature indicates that both are interlaced, with an overall negative impact on the well-being of older people [21]. Mullins [82] interestingly defines loneliness as including both social isolation, i.e., lacking/small social network, and emotional isolation, i.e., lacking persons towards whom one feels affectionate. Other authors [55] found how social isolation and loneliness can be combined in various groups, e.g., neither loneliness nor social isolation, both, and intermediate situations experiencing either social isolation or loneliness.

In the general debate, several studies found that older people without social connection and without a network of confidants, or with a “restricted” network with whom they do not have frequent and in-person relationships, tend to feel alone [71,83,84]. In this respect, Guthmuller [85] highlights that several individual factors in later life are strictly linked with loneliness, e.g., socio-demographic/economic, and regarding health, size of both household and available social resources/network. Also, closeness and frequency of overall contacts, especially when relatives support seniors in their home, have weight. Interestingly, this author also found robust relations between life events which occurred in childhood and loneliness in old age. Other authors [13] highlight that being included in a network of trusting relationships is certainly an antidote to the feeling of loneliness for seniors living alone. This is even though overall broad networks do not seem to reduce the risk of feeling alone more than narrow ones, since the variety of relationships that constitute the overall confidence network is more important. More precisely, a network made up of relatives, friends, neighbours and acquaintances is more effective in containing the level of loneliness compared to a network made up solely of family members, since the former allows seniors to express a wider and more diversified range of confidences.

Further literature underscores how people “can be socially isolated without necessarily feeling lonely and conversely people may feel lonely despite having a broad social network and regular contacts” [21] (p. 5). Similarly, Guthmuller [85] (p. 3) states that “while social isolation and loneliness are strongly associated, lonely persons are not necessarily socially isolated, and persons with a small social network do not necessarily feel lonely”. Other authors confirm such a possible context, i.e., a poor social network does not necessarily mean feeling lonely, since loneliness can exist even when a senior is “surrounded” by several persons [22,86,87]. Stavrova et al. [88] in particular found that spending time with other persons is not related to decreased feelings of loneliness, since for participants reporting severe levels of these feeling, the opportunity to have social contacts with others was associated even with lower well-being than being alone. Seniors could thus not need large social networks, but however require high-quality relationships buffering the feeling of loneliness to promote their overall well-being [89].

It is however important to highlight that, for all respondents, frequent phone calls with relatives and other persons are overall reported as being very important to be less isolated and to feel less alone. Other authors support this finding and indicate that it is possible to have significant and satisfactory relationships with a simple telephone call [90]. However, further studies found that phone calls (and e-mail contacts) do not have “emotional equivalence to the gold standard of embodied presence” [74] (p. 1209), and thus communication mediated by technology “does not match up to older people’s expectations concerning family relationships in later life” [74] (p. 1209).

### 4.4. Social Support Resources, Isolation and Loneliness: Possible Risks for Ageing in Place

The overall combination/synthesis of our findings, with those from the literature, provides a critical picture of frail seniors. The availability and composition of the overall care networks can influence both social isolation, as an objective lack/reduction in relationships and confidants, and the sense of loneliness, as a subjective perception of feeling alone and neglected [30]. The presence of a family seems to have a fundamental and positive role in determining the well-being of older people [31], but also in preventing their institutionalisation and allowing them to age at home [91]. Adult children, when existing/present, can provide in particular the most important support and social contacts in old age, through physical, financial and emotional support to their parents [62]. Neighbours and friends in particular take on the role of primary caregivers when family members are not available [92], and thus act as an important “safety net” [93]. As a result, a lack of supportive family members, friends and neighbours [94], poor availability of public care services [95], in addition to high costs of some private services [96] and possible cultural issues with a PCA [36], could all negatively impact the loneliness and isolation of the old person cared for. This could in turn compromise the possibility of ageing in one’s own home with adequate assistance, and even potentially mutate the home itself into a place of abandonment [97] and potential self-neglect [98].

### 4.5. Possible Policy Interventions

In Europe, the incidence of loneliness and isolation is expected to increase due to population ageing and to individuals living longer [21]. Older people are overall more exposed to the risk of social isolation and loneliness due to the greater likelihood of having to face situations such as living alone, the loss of family and friends, multimorbidity and reduced mobility, with severe consequences especially on mental health [99]. Also, social isolation and loneliness in older people negatively impact their overall well-being [100]. It is thus extremely important to design and implement adequate interventions in this respect.

The overall social protection network of frail older people in Italy, generally (but not always) counteracts social isolation and loneliness, and it is still mainly composed by relatives, as emerged from the IN-AGE study and the previous literature, even though other supports play a role in this respect (PCAs, domestic home helpers, friends/neighbours, services). To support ageing in place it is therefore necessary to identify new strategies/care solutions, with new regulatory, legislative and financing interventions, to strengthen the overall coordination of available services and resources, in order to promote social innovation [101]. Firstly, a collaboration/integration between formal and informal, public and private LTC home services could be more effective as a general starting point. This includes, for instance, PCAs into the formal care system, in addition to respite services, day care centres and training opportunities for family caregivers of seniors living alone. Public health initiatives could enhance social support by promoting community programs/activities to facilitate the social engagement/participation/integration of older people, thus preventing/reducing social disconnectedness and loneliness. These are indeed important aspects impacting anxiety and depression and overall well-being in later life [89,102]. In this respect, it is also worthy to consider how the use of antidepressants can slow the ability of seniors to perform their daily activities, e.g., with increased chance of falling [103], and/or to perceive loneliness, e.g., by dulling emotions [104]. This further stresses the importance of considering loneliness and social isolation when treating older people taking five or more medications (polypharmacy), including antidepressants [105]. Also, some authoritative recommendations [106,107] for strengthening social and psychological assistance, to combat loneliness and social isolation, highlight the concern for the mental health of the population, with a crucial impact on LTC, especially following the COVID-19 pandemic. The resilience of the healthcare system in fact requires the strengthening of primary healthcare and mental health services, to minimise any delays in treatment, especially for older people [108].

Moreover, actions to facilitate innovation with the support of new technologies are needed (e.g., eHealth), since these can improve the efficiency and quality of services, and potentially reduce the isolation and loneliness of older people. Digital devices can help them to remain socially connected to their community [109], thus facilitating their communication with family and friends, as well as giving them the opportunity to access information online [30]. Technology could indeed be a key source to offer new relational/socialisation opportunities and social inclusion, even in extreme conditions of isolation and loneliness in older people [110]. Digital communication tools in particular (smartphone, personal computer, tablet) represent a means of maintaining and increasing the network of social relationships even remotely [60]. However, in Italy a grey digital divide, that is, a gap in digital knowledge between people aged 65–74 and 16–64 years [111], needs to be addressed and managed. Technology could further allow online cognitive–behavioural therapy to mitigate loneliness [112], and providing overall Internet access may have a positive impact on the quality of life of people [113]. It is however important to consider that on the one-side initiatives which use modern technologies can support isolated older people, thus increasing their communication and integration. On the other side, digital interaction reduces human contacts, thus potentially creating further isolation and loneliness. A combination of virtual/in person contacts may be better [21].

It also seems important to enrich, as much as possible, the relational network of seniors, and to expand it beyond the family, through face-to-face presences. These may even be short-term contacts but should guarantee continuity, e.g., more social workers and social operators supporting ideally on a daily basis, in addition to the important support from voluntary associations [13]. Importantly, different combinations/degrees of social isolation/loneliness suggest providing tailored (rather than standardised) interventions to specific needs and functional limitations of senior users [22,114], since “there is no one-size-fits-all approach to addressing loneliness or social isolation” [115] (p. 1). Finally, interventions could include planning and implementing campaigns to raise awareness on these crucial issues, also creating friendship clubs, thus facilitating the design and construction of age-friendly communities [116].

### 4.6. Limitations

This study has some limitations to be considered. The authors did not recruit seniors with cognitive impairments, in order to have participants able to answer questions without help. Moreover, they assessed the cognitive status of seniors with the support of recruitment channels, relatives and interviewers, without conducting a preliminary screening by means of a cognitive test. The definition of frail people was simplified/limited (older persons aged 65 years and over, with functional limitations, living alone and needing support for performing daily activities). This was in order to have a more accessible analysis of frailty [67], since a more holistic and comprehensive approach (including further domains, e.g., low income, multimorbidity, physical activity) [7] is rather complex to address [117,118]. The authors did not assess social support, social isolation and loneliness by means of a validated scale, but only with general/ad hoc open questions. Also, the dimension of social isolation focused only on family members to whom they could confide any intimate concerns/needs, within one’s overall network (including friends and neighbours) and overall face-to-face contacts. The analysis focused on supports only and did not consider possible mediating factors that could affect how older persons perceive their confidants and the quality of help they sought and received. For example, a senior’s ability to seek supports and social connection, through comfortable communication and self-awareness of needs, may impact the level of loneliness a senior feels. To this purpose, such major contributing factors to a senior’s sense of loneliness and/or social isolation could be considered. Another variable that potentially could be addressed, as impacting (and probably increasing) both the perceptions of loneliness and social isolation, is grieving; for instance, following the death of a spouse. Regional and urban/rural comparisons are not carried out, in order to provide first a national picture of the topic, this however hampering the detection of possible geographical discrepancies implying more tailored interventions. Moreover, the authors carried out this study only in three Italian regions, which are however representative of different socio-economic development levels in Italy (Lombardy high; Marche medium; Calabria low) [119]. The findings of this study come from a purposive sample that the authors selected to have a typological, rather than statistical, representativeness. Thus, the findings can only have a theoretical generalisation, as overall contribution to a debate on the explored issues [120]. Furthermore, the authors did not provide a more in-depth quantitative analysis of the findings, also considering statistical values such as *p* and SD values (respectively, significance level and standard deviation), even though this could enrich the study with additional and more punctual considerations. Similarly, the authors did not use the standardised ADL and IADL scales in their entirety and with further additions, and thus they do not have respective reliability and validity values from their study to compare with the literature. Finally, percentage values in the tables should be interpreted with caution, since corresponding absolute values are sometimes very small. Despite these limitations, the authors assured the trustworthiness, in particular of the qualitative analysis, by careful preliminary literature review, accurate description of the study protocol (data collection and analysis process), frequent/collaborative peer de-briefing sessions among researchers and interviewers in all steps of the study. Also, they carried out dissemination seminars with several stakeholders and experts in the field, in order to discuss and validate the findings which they gradually/increasingly collected. This could represent a strength of the study itself and provide insights for further investigations on the topic. However, they did not also provide member verification, i.e., further validation by senior respondents [121].

## 5. Conclusions

In our study, the family emerged as the key “shoulder” for frail older people ageing in place alone in Italy, for both material and social/psychological support and for intimate confidences, especially when the caring relatives and the seniors cared live geographically close. All this is beneficial for experiencing less social isolation and perceive a lesser feeling of loneliness, though not always. Family confidants are however less available when help comes from friends/neighbours and/or public services, especially the latter. Moreover, ageing often brings with it the rarefaction of the support network and more generally of social resources. This is either for “physiological” reasons (loss of a spouse, brothers and sisters, friends), or because physical limitations and reduced mobility make it difficult to maintain and build social bonds. As a result, seniors may feel a sense of abandonment and loneliness; however, at a different degree, and not always associated with a lack of confidants. Policymakers could collaborate with governments, civil society, professionals, stakeholders and the private sector in order to develop and implement adequate policies, also by integrating those already adopted, and by providing appropriate adaptations to local contexts. Such a collaboration could be effective to prevent, manage and overall contrast the social isolation and loneliness of older people [21,114], thus improving their lives, including those of their respective families and communities. In particular, the exchange of national programmes to reduce the social isolation and loneliness of older people as good practices could be encouraged, as is also recommended by the World Health Organization [116] and reported by some authors [21]. In this respect, the following could be mentioned as examples: MONALISA (France), focusing on facilitating contacts among seniors, professionals, family members and volunteers; LinkAge (UK), a platform allowing for connection and knowledge sharing among seniors; Rządowy Programme (Poland), promoting social activities of older people with physical disabilities; KOMP (Norway), a user-friendly digital device (not requiring digital skills) connecting seniors and respective relatives/friends. Future research should however aim to distinguish for whom and how a possible intervention could be operative [115], and should also identify the mediators and moderator factors that affect the various supports older people receive, in addition to their effects on perceived loneliness and social isolation. Other future research could also consider the importance of intergenerational programs as a possibility for older people to interact with younger generations and thus ageing in place more actively and less alone [122,123,124].

## Figures and Tables

**Table 1:** Sample characteristics (absolute values/n and %).

Characteristics	n = 120	%
Age Groups (years)		
67–74	17	14
75–79	19	16
80–84	28	23
85 and over	56	47
Gender		
Male	30	25
Female	90	75
Education		
No title	14	11
Primary–Middle school (5 and 3 years)	75	63
High School–University (3–5 years both)	31	26
Marital Status		
Single/Divorced	32	27
Widowed	88	73
Living Situation		
Alone	93	78
With personal care assistant (PCA)	27	22
Level of physical limitations ^1^		
Mild	30	25
Moderate	33	28
High	27	23
Very high	30	24
Mobility		
Only/Mainly in the home ^2^	48	40
Also outside the home with help ^3^	72	60
Total cases/respondents	120	100

^1^ The level of physical/functional limitations is based on 12 ADLs-IADLs, two mobility limitations (going up/down the stairs and bending to pick up an object), plus sensory limitations in hearing and seeing. Mild = no activities “not able”, Moderate = one-two, High = three-four, Very high = five or more; ^2^ This also includes respondents able to move outside the home very rarely, i.e., less than two times a week and only if accompanied or with aids (cane, walker); ^3^ Respondents are able to move within the home and also outside at least two times a week, only if accompanied or with aids (cane, walker).

**Table 2:** Types of supports/resources (at least one type) ^1^.

Types of Support ^2^	Total	
	n	%
Family	94	78
Children	71	60
Friends/neighbours	50	42
Private services	50	42
Public services	43	36
PCAs	27	23
Total respondents	120	100

^1^ The values in the table do not concern the number of family members, friends, etc., who help, but the number of older persons who reported at least one help of each respective type (one case with family helping = even if with more family members help); ^2^ More types of help/support are possible.

**Table 3:** Network of family confidants and share of family support ^1^.

Family Confidants ^2^	No Family Support		Minority of Family Support		Majority of Family Support		Total	
	n	%	n	%	n	%	n	%
No confidant at all	9	35	2	4	-	-	11	9
No family confidant	10	38	14	27	7	17	31	26
Minority of family confidants	3	12	16	31	8	19	27	23
Majority of family confidants	1	4	8	15	15	36	24	20
Only/all family confidants	3	12	12	23	12	29	27	23
Total respondents	26	100	52	100	42	100	120	100

^1^ Share of family support/help as a proportion of total (from family, private services, public services, PCAs, friends/neighbours). Minority of family support when <50% of total, majority when >50% of total; ^2^ minority of family confidants when <50% of total (from family, friends/neighbours), majority when >50% of total.

**Table 4:** Network of family confidants and living close relatives who support ^1^.

Family Confidants ^2^	No Close Family Support		One Close Family Support		Two/More Close Family Supports		Total	
	n	%	n	%	n	%	n	%
No confidant at all	-	-	1	4	1	3	2	2
No family confidant	12	35	7	27	2	6	21	22
Minority of family confidants	9	26	8	31	7	21	24	26
Majority of family confidants	7	21	3	12	13	38	23	24
Only/all family confidants	6	18	7	27	11	32	24	26
Total respondents	34	100	26	100	34	100	94	100

^1^ Family members living close who help, i.e., who live in the same urban city/rural municipality of respondents; ^2^ Minority of family confidants when <50% of total (from family, friends/neighbours), majority when >50% of total.

**Table 5:** Face-to-face contacts and relatives living close who support ^1^.

Face-to-Face Contacts ^2^	No Close Family Support		One Close Family Support		Two/More Close Family Supports		Total	
	n	%	n	%	n	%	n	%
No confidant at all	-	-	1	4	1	3	2	2
No face-to-face confidant	4	12	1	4	-	-	5	5
1 face-to-face confidant	10	29	4	15	6	18	20	21
2 face-to-face confidants	12	35	10	38	10	29	32	34
3+ face-to-face confidant	8	24	10	38	17	50	35	37
Total respondents	34	100	26	100	34	100	94	100

^1^ Family members living close who help, i.e., who live in the same urban city/rural municipality of respondents; ^2^ Face-to-face contacts with one’s confidants in general (not only family members).

**Table 6:** Network of family confidants and other supports (at least one type of help) ^1^.

Family Confidants ^2^	Public Services		Private Services		Friends/Neighbours		PCA		Total	
	n	%	n	%	n	%	n	%	n	%
No confidant at all	8	19	7	14	2	4	1	4	11	9
No family confidant	17	40	10	20	20	40	4	15	31	26
Minority of family confidants	5	12	11	22	14	28	10	37	27	23
Majority of family confidants	3	7	10	20	6	12	8	30	24	20
Only/all family confidants	10	23	12	24	8	16	4	15	27	23
Total respondents	43	100	50	100	50	100	27	100	120	100

^1^ More types of help/support are possible; ^2^ minority of family confidants when <50% of total (from family, friends/neighbours); majority when >50% of total.

**Table 7:** Perceived loneliness and share of family support ^1^.

Loneliness Levels ^2^	No Family Support		Minority of Family Support		Majority of Family Support		Total	
	n	%	n	%	n	%	n	%
Absent/mild	6	23	16	31	17	40	39	33
Moderate	7	27	13	25	7	17	27	23
High	7	27	15	29	9	21	31	26
Very high	6	23	8	15	9	21	23	19
Total respondents	26	100	52	100	42	100	120	100

^1^ Share of family support/help as a proportion of total (from family, private services, public services, PCAs, friends/neighbours). Minority of family support when <50% of total, majority when >50% of total; ^2^ Loneliness: absent/mild if the person does not/rarely feel alone; moderate if the person feels sometimes lonely; high if the person often feels lonely; very high if the person often feels alone with depressive states and insomnia.

**Table 8:** Perceived loneliness and living close relatives who support ^1^.

Loneliness Levels ^2^	No Close Family Support		One Close Family Support		Two/More Close Family Supports		Total	
	n	%	n	%	n	%	n	%
Absent/mild	13	38	7	27	13	38	33	35
Moderate	7	21	9	35	4	12	20	21
High	10	29	6	23	8	24	24	26
Very high	4	12	4	15	9	26	17	18
Total respondents	34	100	26	100	34	100	94	100

^1^ Family members living close who help, i.e., who live in the same urban city/rural municipality of respondents; ^2^ Loneliness: absent/mild if the person does not/rarely feel alone; moderate if the person sometimes feels lonely; high if the person often feels lonely; very high if the person often feels alone with depressive states and insomnia.

**Table 9:** Perceived loneliness and other supports (at least one type of help) ^1^.

Loneliness Levels ^2^	Public Services		Private Services		Friends/Neighbours		PCA		Total	
	n	%	n	%	n	%	n	%	n	%
Absent/mild	9	21	13	26	13	26	14	52	39	33
Moderate	11	26	15	30	16	32	3	11	27	23
High	13	30	12	24	13	26	7	26	31	26
Very high	10	23	10	20	8	16	3	11	23	19
Total respondents	43	100	50	100	50	100	27	100	120	100

^1^ More types of help/support are possible; ^2^ Loneliness: absent/mild if the person does not/rarely feel alone; moderate if the person feels sometimes lonely; high if the person often feels lonely; very high if the person often feels alone with depressive states and insomnia.

**Table 10:** Network of family confidants and perceived loneliness ^1^.

Family Confidants ^2^	Absent/Mild		Moderate		High		Very High		Total	
	n	%	n	%	n	%	n	%	n	%
No confidant at all	2	5	4	15	2	6	3	13	11	9
No family confidant	8	21	8	30	7	23	8	35	31	26
Minority of family confidants	11	28	3	11	8	26	5	22	27	23
Majority of family confidants	9	23	6	22	5	16	4	17	24	20
Only/all family confidants	9	23	6	22	9	29	3	13	27	23
Total respondents	39	100	27	100	31	100	23	100	120	100

^1^ Loneliness: absent/mild if the person does not/rarely feel alone; moderate if the person sometimes feels lonely; high if the person often feels lonely; very high if the person often feels alone with depressive states and insomnia; ^2^ Minority of family confidants when <50% of total (from family, friends/neighbours), majority when >50% of total.

## Data Availability

Some quantitative data presented in this study are openly available in Mendeley at https://doi.org/10.17632/3ryrpz224h.2 (accessed on 5 March 2024). Not all quantitative data presented in the paper are available (e.g., loneliness and social isolation), since sensitive and potentially identifying information, when in combination with basic characteristics of the respondents. Also, the charts with all original verbatim transcriptions are not publicly available due to privacy/ethical restrictions, which due to them containing information that could compromise the privacy/anonymity of the research participants (e.g., include names of persons and locations and other potential identifiers of respondents).
